# The SET Complex Acts as a Barrier to Autointegration of HIV-1

**DOI:** 10.1371/journal.ppat.1000327

**Published:** 2009-03-06

**Authors:** Nan Yan, Peter Cherepanov, Janet E. Daigle, Alan Engelman, Judy Lieberman

**Affiliations:** 1 Immune Disease Institute and Department of Pediatrics, Harvard Medical School, Boston, Massachusetts, United States of America; 2 Division of Medicine, Imperial College London, St. Mary's Campus, London, United Kingdom; 3 Department of Cancer Immunology and AIDS, Dana-Farber Cancer Institute, Boston, Massachusetts, United States of America; Aaron Diamond AIDS Research Center, United States of America

## Abstract

Retroviruses and retrotransposons are vulnerable to a suicidal pathway known as autointegration, which occurs when the 3′-ends of the reverse transcript are activated by integrase and then attack sites within the viral DNA. Retroelements have diverse strategies for suppressing autointegration, but how HIV-1 protects itself from autointegration is not well-understood. Here we show that knocking down any of the components of the SET complex, an endoplasmic reticulum-associated complex that contains 3 DNases (the base excision repair endonuclease APE1, 5′-3′ exonuclease TREX1, and endonuclease NM23-H1), inhibits HIV-1 and HIV-2/SIV, but not MLV or ASV, infection. Inhibition occurs at a step in the viral life cycle after reverse transcription but before chromosomal integration. Antibodies to SET complex proteins capture HIV-1 DNA in the cytoplasm, suggesting a direct interaction between the SET complex and the HIV preintegration complex. Cloning of HIV integration sites in cells with knocked down SET complex components revealed an increase in autointegration, which was verified using a novel semi-quantitative nested PCR assay to detect autointegrants. When SET complex proteins are knocked down, autointegration increases 2–3–fold and chromosomal integration correspondingly decreases ∼3-fold. Therefore, the SET complex facilitates HIV-1 infection by preventing suicidal autointegration.

## Author Summary

When HIV-1 infects a cell, its genomic RNA is copied into DNA. The ends of the viral DNA are then activated by the viral integrase enzyme to enable DNA insertion into a host cell chromosome. However, the activated ends can alternately insert into the virus itself by a process called autointegration, which is a suicidal pathway that aborts the infection. How HIV-1 protects itself from suicidal autointegration is not known. Here we show that a cytoplasmic complex, called the SET complex, which contains three DNA digesting enzymes, binds to HIV-1 and protects it from autointegration.

## Introduction

Soon after HIV-1 enters a susceptible target cell, the viral genomic RNA is reverse transcribed within the reverse transcription complex (RTC) to double-stranded DNA [1]. The RTC matures into the preintegration complex (PIC), which delivers the viral DNA to the nucleus for integration into a chromosome [2]. The PIC may also sequester and protect the viral DNA from cellular DNA-modifying enzymes [3] and from cytoplasmic DNA sensors [4]–[6] that could trigger antiviral innate immunity. Surprisingly little is known about the host proteins that associate with the PIC and assist in HIV-1 integration. Integration can be divided into three steps: (1) 3′ processing (integrase (IN)-mediated hydrolysis of GT dinucleotides from HIV-1 DNA to produce reactive, recessed CA_OH_-3′ ends); (2) DNA strand transfer (IN-mediated insertion of the cleaved 3′ ends into opposing strands of host chromosomal DNA); and (3) 5′-end joining (repair by host enzymes of the gaps between the 5′-ends of viral DNA and the chromosome) [7].

3′-processing makes the viral DNA vulnerable to autointegration [8],[9] in which the reactive CA ends attack sites within the viral DNA. Autointegration is mechanistically analogous to chromosomal integration, but results in nonproductive deletion or inversion circles [9]–[12]. Autointegration is a problem faced not only by retroviruses, but also by mobile genetic elements including bacteriophages and retrotransposons [10],[13],[14]. Each element employs a unique mechanism, relying on either self or host factors, to control autointegration. For example, bacteriophage Mu B protein activates DNA strand transfer to favor intermolecular transposition [14],[15]. In the case of Tn10, a cellular global regulator, H-NS, acts directly on the PIC to promote intermolecular transposition [16]. The barrier-to-autointegration factor (BAF) is a cellular protein that protects Moloney murine leukemia virus (MLV) PICs from autointegration and stimulates intermolecular integration in vitro [12],[17]. Although BAF can also stimulate HIV-1 PIC intermolecular integration activity in vitro, it has not been shown to block HIV-1 autointegration [18]–[20].

3′-processing can occur soon after the DNA ends are synthesized by reverse transcription in the cytoplasm [21], suggesting that a cytoplasmic mechanism might be needed to protect HIV-1 from autointegration. We therefore considered host cytosolic DNA-interacting proteins as potential regulators of autointegration. One candidate is the SET complex, an endoplasmic reticulum (ER)-associated DNA repair complex that contains three DNases and is mobilized to the nucleus in response to oxidative stress. The SET complex was discovered as a Granzyme A (GzmA) target in cells undergoing caspase-independent T cell-mediated death [22]. Two nucleases in the complex, the endonuclease NM23-H1 and the exonuclease TREX1, are activated by GzmA cleavage of the inhibitor SET protein to cause single-stranded DNA damage [23],[24]. In addition to the three DNases (APE1, NM23-H1, TREX1) and SET (a histone chaperone of the nucleosome assembly protein family), the SET complex contains HMGB2, a DNA binding protein that preferentially binds to distorted or damaged DNA, and the PP2A inhibitor pp32 [25]. Although individual SET complex components have been implicated in diverse processes (including DNA repair, histone modification, DNA replication, transcriptional activation, single-stranded DNA degradation, autoimmunity), the functions of the intact complex are not well understood [26]. Here we show that the SET complex plays an important role in the early phase of the HIV-1 lifecycle by inhibiting autointegration.

## Results

### Silencing SET complex proteins inhibits HIV-1 infection

**Figure 1:**
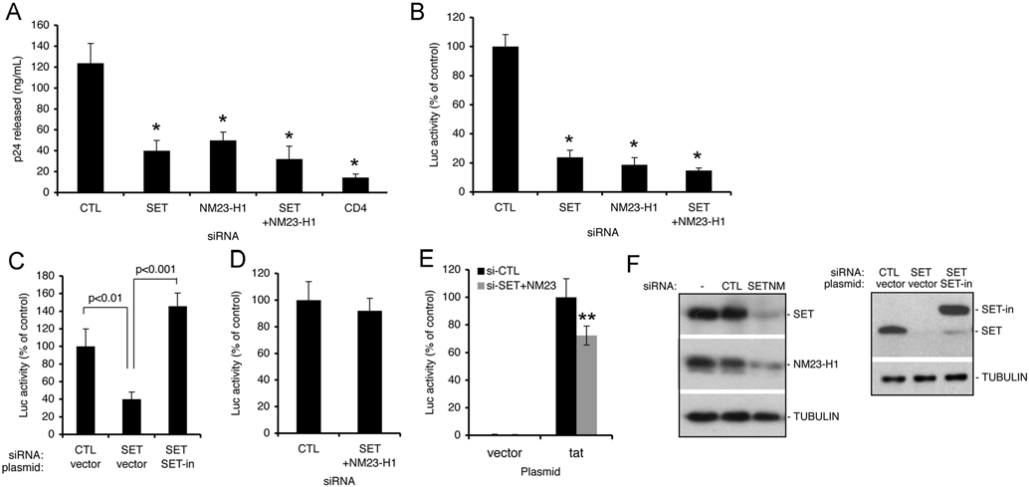
The SET complex facilitates HIV-1 infection. (A) SET and/or NM23-H1 knockdown inhibits HIV-1_IIIB_ infection. HeLaCD4 cells transfected with a non-targeting control siRNA (CTL) or siRNAs targeting SET and/or NM23-H1 were infected with HIV-1_IIIB_, and infection was assayed by p24 release. (B) SET and/or NM23-H1 knockdown blocks single-round HIV-Luc infection. HeLaCD4 cells were transfected with indicated siRNAs and infected with VSV-G pseudotyped HIV-Luc. Luciferase (Luc) activity, measured 48 hpi and normalized to total cellular protein, is compared to Luc activity in cells transfected with control siRNA. (C) Expression of siRNA-insensitive SET (SET-in) rescues the HIV-Luc infection block caused by knocking down endogenous SET. 293T cells, transfected with control or SET siRNAs and then transfected two days later with empty vector or SET-in and pCMV-β-gal plasmids, were infected with HIV-Luc 24 h after transfection, and Luc activity measured 48 h later was normalized to β-galactosidase activity. (D) Expression from transfected HIV-Luc DNA is not affected by SET/NM23-H1 knockdown. HeLaCD4 cells were transfected with HIV-Luc plasmid two days following siRNA transfection. Luc activity was measured 24 h later and normalized as in (D). (E) Expression from a chromatinized HIV-Luc reporter gene is weakly affected by SET/NM23-H1 knockdown. TZM-bl cells that harbor an integrated LTR-Luc reporter gene were first transfected with control or SET/NM23-H1 siRNA, and then transfected 48 h later with either an empty vector or a Tat expression plasmid to activate reporter gene expression. Luc activity was measured 24 h later. (F) Immunoblots showing SET/NM23-H1 knockdown and SET-in over-expression. SET-in is an siRNA-insensitive FLAG-HA-tagged protein. *, p<0.001 relative to control knockdown in (A) and (B). **, p<0.05 relative to control in (E). Mean and standard deviation (S.D.) from at least four independent infections are shown in (A–E).

Knockdown of SET and/or NM23-H1 in HeLaCD4 cells reduced HIV-1_IIIB_ infectivity 3 to 4-fold as assessed by p24 levels in culture supernatants (Figure 1A). Although viral replication was impaired, the virions produced from knockdown cells were equally infectious when applied in equivalent amounts (normalized by p24 level) to indicator TZM-bl cells that express an HIV LTR-driven luciferase (Luc) reporter gene (Figure S1). This suggested that SET and NM23-H1 act early in the viral life cycle. To focus on early events, cells were infected with an HIV-1-derived single-round reporter virus (HIV-Luc) pseudotyped with the vesicular stomatitis virus G (VSV-G) envelope glycoprotein [18], and infection was assessed two days later by Luc activity. Knockdown of SET, NM23-H1, or both reduced Luc activity to 24%, 19%, and 15% of control levels, respectively (Figure 1B). HIV-Luc activity was restored by expressing RNAi-insensitive SET (SET-in) in SET siRNA-treated cells (Figure 1C). SET knockdown and SET-in rescue had similar effects when infections were performed using a range of multiplicities of infection (MOI) (Figure S2). Single-round virus carrying the natural HIV-1 envelope glycoprotein was similarly inhibited by SET and/or NM23-H1 knockdown (data not shown).

Because inhibition was independent of the envelope glycoprotein, SET and NM23-H1 likely influenced post-entry steps. SET can influence chromatin accessibility in its role as a histone chaperone and inhibitor of histone acetylation and DNA demethylation, and NM23-H1 enhances the transcription of some genes [26],[27]. We therefore tested whether knocking down SET and NM23-H1 inhibited transcription from transfected HIV-Luc plasmid DNA and from a chromatinized reporter gene. There was no significant effect on expression from transfected HIV-Luc and only a weak effect (∼25%, p<0.05) on Tat-dependent expression from the chromatinized reporter gene (Figure 1D and 1E). Together these experiments suggest that SET and NM23-H1 act primarily downstream of viral entry and before Tat-dependent transcription.

**Figure 2:**
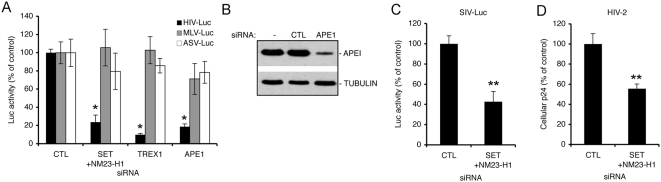
Knockdown of SET complex proteins inhibits HIV-1 and HIV-2/SIV, but not MLV or ASV infection. (A) Knockdown of SET/NM23-H1, APE1, or TREX1 inhibits HIV-Luc, but not MLV or ASV single-round reporter viruses. Luc activity was measured 48 hpi. (B) Immunoblot confirms APE1 knockdown. TREX1 knockdown, assayed by qRT-PCR, reduced TREX1 mRNA by 94% (not shown). (C,D) Knockdown of SET/NM23-H1 also inhibits SIV-Luc (C) and HIV-2 (D) infection. *, p<0.01. **, p<0.05.

The SET protein is a component of at least two complexes – a ∼150 kDa nuclear complex that contains SET and pp32 and some of their paralogues and the ∼270–420 kDa ER-associated SET complex [22],[28]. Because NM23-H1 knockdown interfered with HIV-1, we reasoned that the larger SET complex facilitated infection. In fact, knocking down two other members of this complex, APE and TREX1, similarly reduced HIV-Luc activity (Figure 2).

To test whether SET complex proteins enhance infection of other retroviruses, the effect of knocking down SET complex proteins on MLV and avian sarcoma virus (ASV) was tested using similar Luc reporter systems. Both MLV-Luc and ASV-Luc were largely unaffected when SET complex proteins were knocked down (Figure 2A and 2B). By contrast, the infectivity of two other lentiviruses, SIV-Luc and HIV-2, was reduced approximately two-fold by SET/NM23-H1 knockdown (Figure 2C and 2D). These results suggest that the SET complex specifically affects lentiviral infection.

### SET/NM23-H1 knockdown reduces chromosomal integration

**Figure 3:**
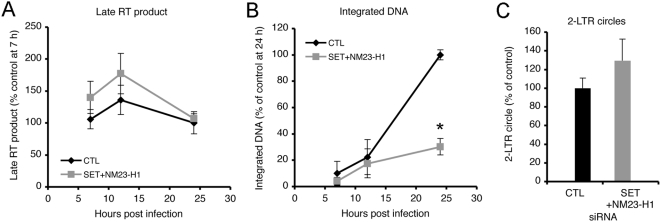
HIV-1 integration is reduced in SET/NM23-H1 knockdown cells. (A–C) Late RT products (normalized to mitochondrial DNA) (A) and integrated DNA (normalized to β-globin sequences) (B), measured at indicated times, and 2-LTR circles, measured 24 hpi (C), from control (black) or SET/NM23-H1 (gray) knockdown cells. Data are mean+/−S.D. of triplicate measurements from three independent experiments, normalized to control knockdown cells. *, p<0.001.

To pinpoint the block in the viral life cycle, we compared the effect of SET/NM23-H1 knockdown on stage-specific HIV-1 DNA products by quantitative PCR (qPCR) [29],[30]. Late reverse transcription products (late RT) measured during the first day of infection were not significantly different in knockdown cells (Figure 3A). In contrast, integrated HIV-1 DNA (quantified by nested Alu-PCR) was reduced by 3-fold 24 h post infection (hpi) in SET/NM23-H1 knockdown cells (Figure 3B). Two-long terminal repeat (2-LTR) circles were slightly (about 28%, p>0.05) increased by the knockdown (Figure 3C).

**Figure 4:**
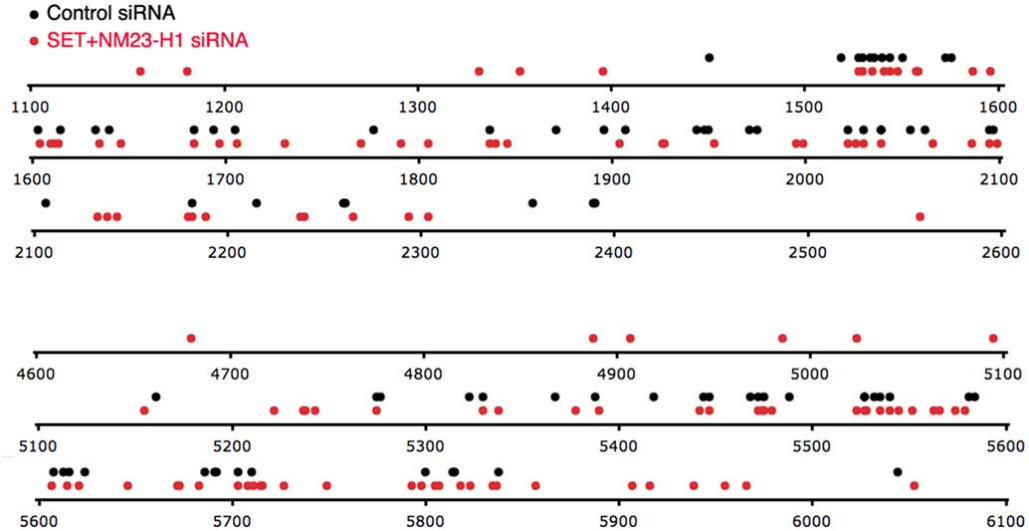
Autointegration sites recovered from control and SET/NM23-H1 knockdown libraries. Each site within selected 1.5-kb regions of the HIV-1 genome (numbering based on the HIV-1_NL4-3_ strain) is represented as a dot. Autointegrants from control siRNA-treated and SET/NM23-H1 knockdown cells are black and red, respectively.

To understand why HIV-1 integration might be impaired, insertion sites were sequenced [29] using DNA isolated 24 hpi from control and SET/NM23-H1 knockdown cells. HIV-1 normally integrates preferentially into transcriptionally active chromatin [31],[32]. The frequency of integration within transcription units, CpG islands, and promoters was not significantly different in the knockdown cells (Table 1). Although the DNA for the integration site analysis was isolated from the Hirt pellet, which is enriched for chromosomal DNA, a significant number of clones arose from autointegration. The proportion of autointegrants recovered from SET/NM23-H1 knockdown cells exceeded the control, comprising 259 of 816 (32%) vs 182 of 816 (22%) sequences (p<0.0001) (Table 1, Figure 4). The autointegration sites in the control and knockdown cells showed the same sequence preference as chromosomal integration, favoring insertion within GG dinucleotides (Figure S3) [29],[33].

**Table 1:** Increased number of HIV autointegration events recovered from SET/NM23-H1 knockdown cells.

	Control Knockdown Library		SET+NM23-H1 Knockdown Library		p-Value (Comparing Two Libraries)
	Number of Sequences	% of Total Unique Integration Sites	Number of Sequences	% of Total Unique Integration Sites	
Total	816		816		
Passed initial parsing	680		623		
Autointegrants	182		259		<0.001
Mapped to genome	294		232		
Unique sites	239	100%	172	100%	
mRNA	204	85.4%	147	85.5%	
Ensembl	198	82.8%	137	79.7%	
Refseq	190	79.5%	132	76.7%	>0.5
CpG-1kb	3	1.3%	0	0%	
CpG-2kb	25	10.5%	11	6.4%	>0.1
CpG-5kb	59	24.7%	40	23.3%	
Refseq-2.5kb	18	7.5%	10	5.8%	>0.6
Refseq-5kb	38	15.9%	29	16.9%	

SET/NM23-H1 knockdown does not affect chromosomal integration site preferences, but enhances autointegration. DNA was cloned and sequenced from the Hirt pellet.

Gene IDs for the genes mentioned in the text are: SET 6418, NM23-H1 4830, APE1 328, TREX1 11277, PP2A 5515, PP32 8125, HMGB2 3148, LEDGF 11168, BAF 8815, and APOBEC3G 60489.

### SET/NM23-H1 knockdown enhances autointegration

**Figure 5:**
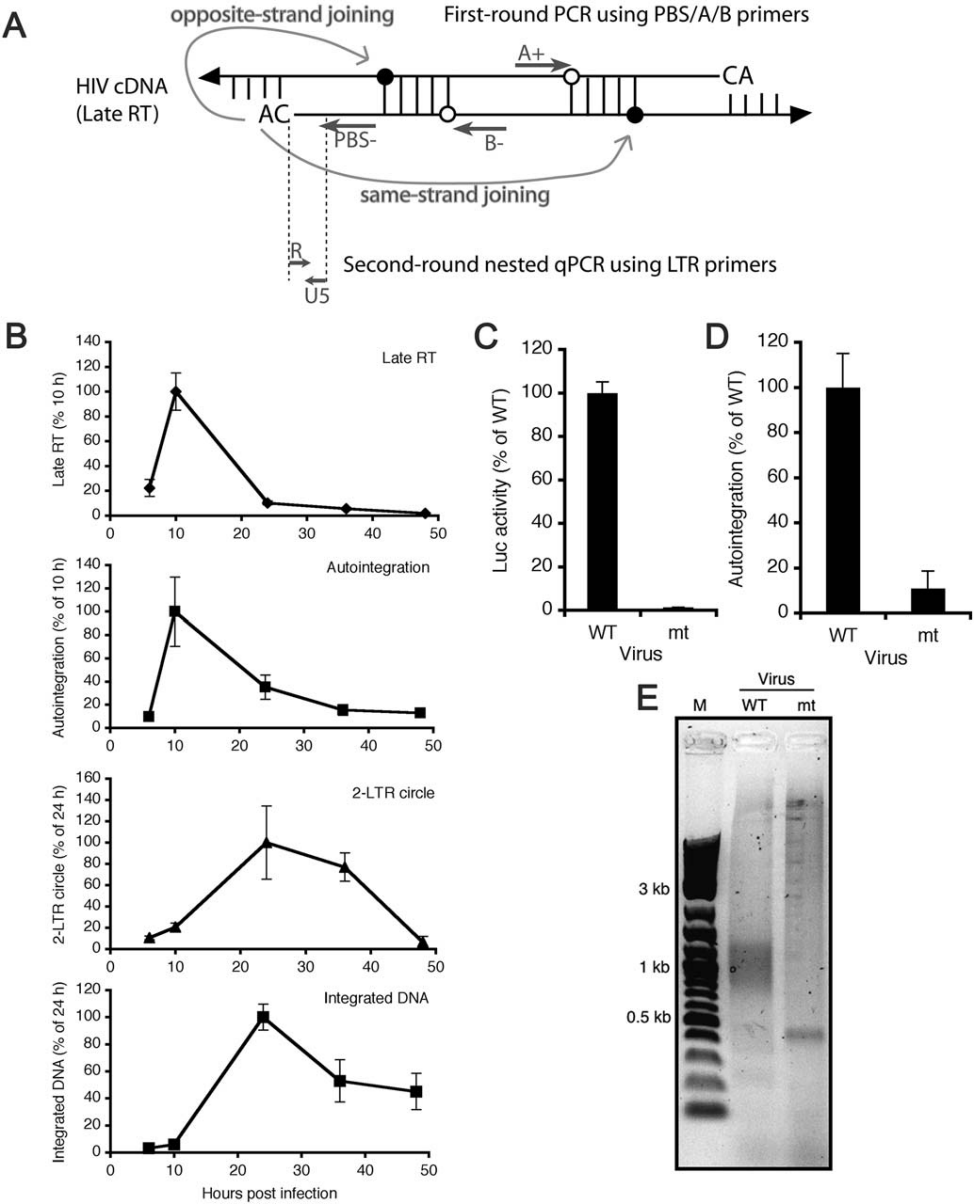
A PCR-based assay for measuring HIV autointegration (auto-PCR). (A) Diagram of HIV-1 auto-PCR assay (a more detailed diagram is provided in Figure S4). The common primer binding site (PBS−) (reverse) primer is used to amplify same strand integrants with the A+ (forward) primer or opposite strand integrants with the B− (reverse) primer during first-round PCR. A single-length nested PCR product is then amplified using an LTR primer pair (R-U5) during second-round qPCR. Filled circles, internal viral 5′ phosphates attacked during autointegration. (B) Kinetics of stage-specific HIV-1 DNA product formation. Late RT, autointegrants, and 2-LTR circles were normalized to mitrochondrial DNA; integrated DNA was normalized to β-globin. Values of late RT and autointegration are shown relative to peak values 10 hpi, while 2-LTR and integrated DNA are normalized to peak 24 hpi values. Mean and S.D. from triplicate qPCR measurements are shown. (C–E) Active IN is required for autointegration. HeLaCD4 cells were infected with HIV-Luc carrying wild type (WT) or mutant (mt) IN. Luc activity was measured 48 hpi (C) and auto-PCR was performed 10 hpi (D). First-round PCR products (E) from Hirt supernatant DNA were analyzed by agarose gel electrophoresis.

Because there are no assays to quantify HIV-1 autointegration, a nested qPCR autointegration assay (auto-PCR) was designed to quantify and clone autointegration events from Hirt supernatant DNA. Three primers (PBS− (primer binding site), A+, and B−) were designed to detect integration of the minus strand U3 CA-3′ end into either strand of viral DNA (Figures 5A and S4). The first PCR round generates products that contain the upstream LTR and internal viral sequences of variable length depending on the distance between the site of autointegration and primer A+ or B−. The qPCR (second) round amplifies LTR sequences of a fixed length from diluted first-round PCR products. To validate the assay, we verified that first-round PCR using only PBS or A+ and B− primers amplified negligible amounts of LTR-containing DNA compared to reactions with all 3 primers. Autointegration is expected to occur shortly after reverse transcription because 3′ processing can happen soon after DNA synthesis [21]. Auto-PCR and late RT DNAs both peaked 10 hpi, while 2-LTR circles and integrated DNA peaked 24 hpi (Figure 5B). These kinetics support the specificity of the auto-PCR assay to detect autointegrants rather than 2-LTR circles. Consistent with the hypothesis that most autointegration events likely arise from the concerted insertion of both U3 and U5 viral DNA ends [34], the kinetics of U5 end joining closely mirrored those of U3 (Figure S5). Since autointegration requires IN activity, HeLaCD4 cells were infected with HIV-Luc carrying either wild-type (WT) or active site mutant (D64N/D116N, mt) IN. As expected, auto-PCR product formation was significantly reduced following mt IN viral infection (Figure 5D). First-round PCR products analyzed by electrophoresis through agarose gels produced a smear migrating at ∼1 kb from WT-infected cells, while mt virus products had no appreciable DNA in this region (Figure 5E). The 1 kb smear is likely the consequence of the 3 min extension time used in the first round PCR (see Materials and Methods). DNA from the regions corresponding to the 1 kb smear were isolated, cloned, and sequenced. 21 of 30 clones from the WT viral infection contained an IN-processed U3 end (identified by loss of the GT dinucleotide from the unprocessed CAGT strand). The processed U3 end joined to an internal viral sequence in 13 cases, whereas the remaining 8 sequences contained only viral LTR sequences (Table S1). Only 5 of 30 clones from the mt IN virus infection contained any viral sequence, and none contained a processed U3 end (Table S1). The mt virus likely supported low level auto-PCR product formation (Figure 5D) due to background amplification of nonspecific first round PCR products (Figure 5E, Table 1) and/or increased levels of unintegrated DNA that form under these infection conditions [35]. These results demonstrate that the auto-PCR assay predominantly amplifies autointegrated HIV-1 DNA.

**Figure 6:**
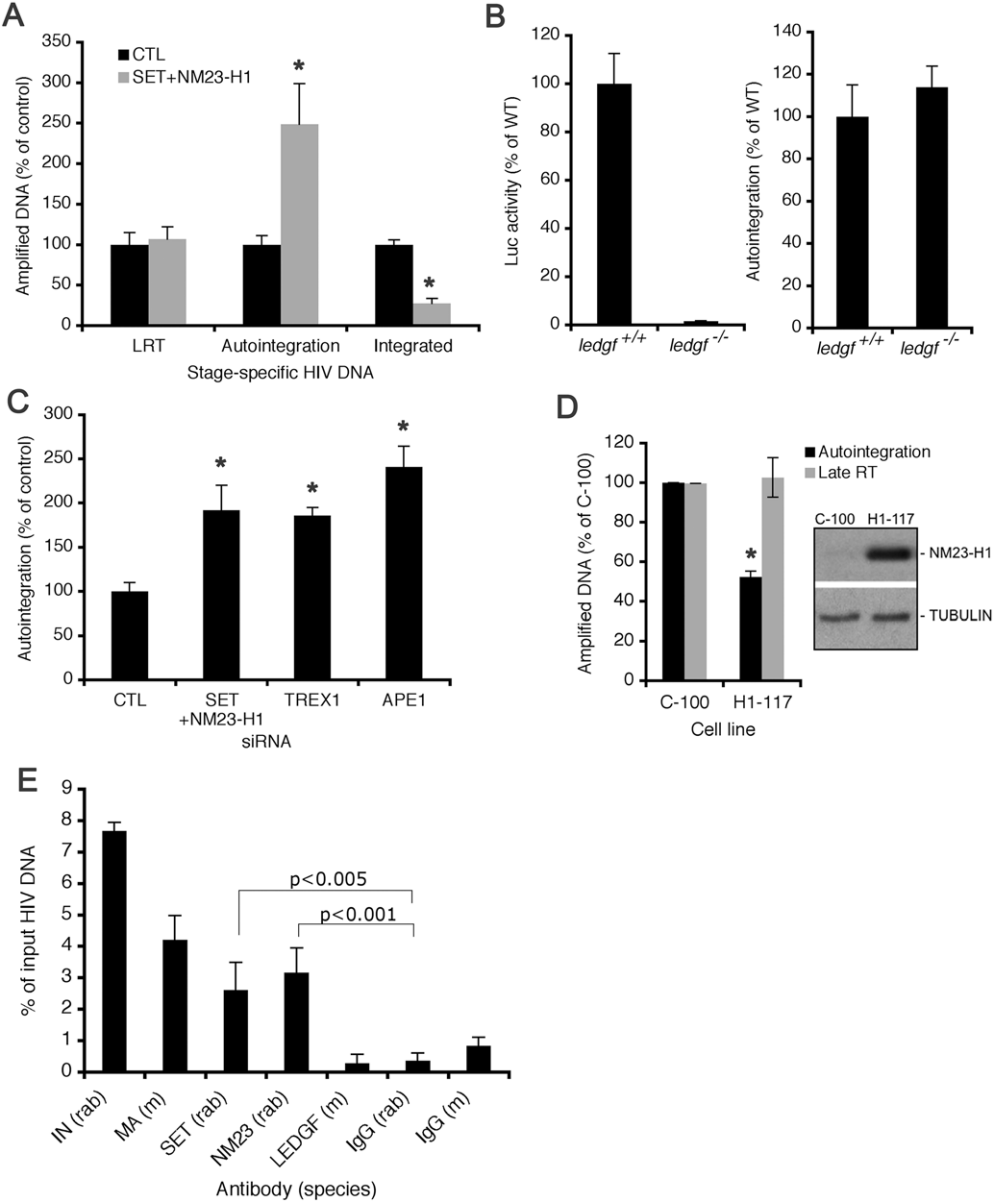
The SET complex suppresses HIV-1 autointegration. (A) Stage specific HIV-1 DNAs from control and SET/NM23-H1 knockdown cells infected with HIV-Luc. Late RT (LRT) and autointegration were measured 10 hpi and chromosomal integration was assayed 24 hpi. Mean and S.D. from triplicate qPCR assays of three independent experiments are shown. *, p<0.01. (B) Autointegration is not an obligate by-product of failed integration. Autointegration was measured 10 hpi and Luc activity at 48 hpi. The difference in autointegration is not significant. (C) Knocking down other SET complex proteins, TREX1 or APE1, also increases autointegration. Mean plus S.D. from two independent experiments are shown. *, p<0.01. (D) NM23-H1 over-expression suppresses autointegration. MDA-MB-435, an NM23-H1–deficient metastatic breast cancer cell line, stably transfected with vector (C-100) or an NM23-H1 expression plasmid (H1-117), were infected with VSV-G pseudotyped HIV-Luc, and LRT and autointegrants were measured 10 hpi. *, p<0.01. (E) SET and NM23-H1 associate with HIV-1 cDNA. Cytoplasmic extracts from infected HeLaCD4 cells were immunoprecipitated with the indicated antibodies (mouse (m) or rabbit (rab)), and associated HIV-1 cDNA was quantified by qPCR. Mean plus S.D. from three independent experiments are shown.

With the auto-PCR assay validated, we compared autointegration and other stage-specific HIV-1 DNAs in control and SET/NM23-H1 knockdown cells (Figure 6A). Late RT products were comparable 10 hpi as shown in Figure 3A, whereas autointegration assayed at the same time increased 2.5 fold in SET/NM23-H1 knockdown cells (p<0.01). Chromosomal integration measured 24 hpi decreased 3-fold (p<0.001), as expected, in SET/NM23-H1 knockdown as compared to control cells (Figure 6A). The corresponding increase in autointegration and decrease in chromosomal integration suggested that the integration defect is due to reduced available substrate because of suicidal autointegration.

Because increased autointegration occurred before chromosomal integration, it was unlikely that autointegration was secondary to failed chromosomal integration. To test directly whether autointegration might be an obligate side product of failed integration, we quantified autointegration events in *ledgf* ^−/−^ and *ledgf* ^+/+^ mouse embryo fibroblasts (MEF) infected with HIV-Luc. LEDGF is a nuclear factor, which tethers the PIC to genomic DNA and plays a crucial role in chromosomal integration [29],[36],[37]. Although HIV-Luc infection of *ledgf* ^−/−^ cells was barely detectable compared to *ledgf* ^+/+^ MEF, autointegration did not significantly change in *ledgf* ^−/−^ MEF (the IN within the PIC is fully active in *ledgf* ^−/−^ cells [29]) (Figure 6B). Therefore autointegration is not an obligate side effect of decreased chromosomal DNA integration. LEDGF and the SET complex did not coimmunoprecipitate in infected cells and recombinant IN and SET also did not coprecipitate (data not shown). Our results collectively indicate that the SET complex suppresses autointegration rather than augments chromosomal integration. In support of this, individual knockdown of TREX1 or APE1, two other SET complex components, also significantly increased autointegration (Figure 6C). Just as NM23-H1 knockdown enhanced autointegration, overexpressing NM23-H1 in an NM23-H1 defective human breast cancer cell line (MDA-MB-435) suppressed HIV-1 autointegration by 2-fold (p<0.001) but had no effect on reverse transcription (Figure 6D).

BAF can augment HIV-1 integration in vitro [20] and in cells [19], although its overall importance during virus infection is controversial [18],[19]. To determine whether BAF regulates autointegration, lysates prepared from HIV-Luc-infected control and BAF knockdown cells were assayed by auto-PCR. BAF knockdown was efficient (92% protein knockdown) and reduced Luc activity about 2-fold (Figure S6A and S6B), but had no effect on autointegration (Figure S6C). Although we cannot exclude a role for BAF in regulating autointegration, its knockdown reduced HIV-1 infection ∼2-fold without a concomitant change in auto-PCR product formation. We therefore conclude that suppression of autointegration is unlikely to be the dominant mechanism through which BAF regulates HIV-1 infection.

### SET and NM23-H1 associate with HIV-1 DNA in the cytoplasm

Autointegration product formation peaked in parallel with the late RT product (Figure 5B), suggesting that autointegration occurs in the cytoplasm. In fact, 68% of autointegrants at their peak 10 hpi were in cytoplasmic rather than nuclear lysates (data not shown). We therefore predicted that the SET complex would associate with HIV-1 reverse transcripts in the cytoplasm. The ability of SET complex and control antibodies to capture HIV-1 cDNA from cytoplasmic lysates 6 hpi was analyzed (Figure 6E). IN and matrix (MA) antibodies captured 7.7% and 4.3% of cytoplasmic HIV-1 DNA, respectively, as assessed by qPCR. LEDGF antibody did not pull down a significant amount of HIV-1 DNA, in contrast to a previous report [38]. SET and NM23-H1 antibodies, which immunoprecipitated ∼30–40% of these abundant proteins from input samples (data not shown), pulled down 2.4% and 3.1% of HIV-1 DNA, respectively, significantly more than rabbit IgG control (p<0.005 and p<0.001, respectively). The direct association of SET complex proteins with HIV-1 DNA in the cytoplasm early in infection further supports its role in preventing autointegration.

### APE1 enhances HIV replication in Jurkat T cells

**Figure 7:**
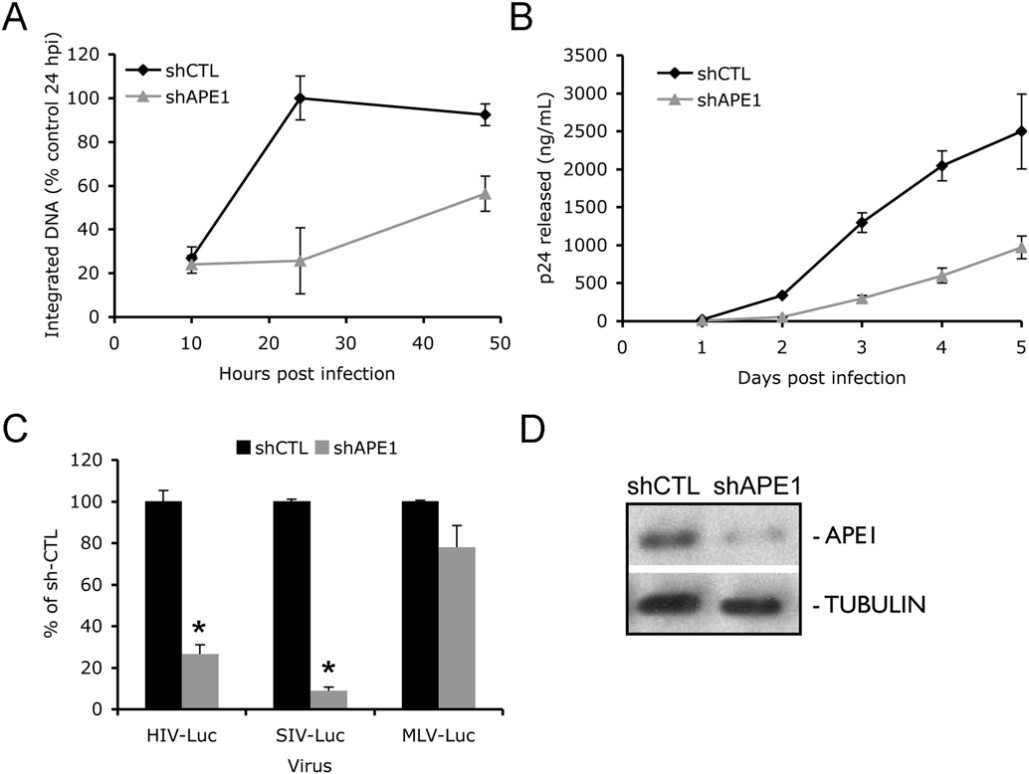
APE1 enhances HIV infection in Jurkat T cells. Jurkat cells expressing sh-CTL or sh-APE1 were infected with HIV_IIIB_, and integrated DNA (A) and p24 release into the medium (B) were measured at indicated times. Mean plus S.D. from two independent experiments are shown. (C) APE1 knockdown in Jurkat cells inhibits HIV-Luc and SIV-Luc, but not MLV-Luc, infectivity. (D) Immunoblot showing APE1 knockdown in sh-APE1 lentivirus-infected Jurkat cells.

Since our previous studies were performed in HeLaCD4 cells, we wanted to verify the postulated role for the SET complex in more physiologically relevant T cells. We produced Jurkat cells stably knocked down for APE1 by infection with a lentivirus expressing a shRNA targeting APE1 (sh-APE1). Knocking down APE1 did not alter cell viability or proliferation (data not shown). APE1 knockdown cells infected with HIV_IIIB_ had significantly reduced levels of integrated HIV DNA and viral production compared to cells expressing the sh-CTL control hairpin (Figure 7A and 7B), suggesting that APE1 also facilitates HIV-1 infection in immune system cells. SIV-Luc infection was also strongly inhibited (∼10-fold) in Jurkat cells by APE1 knockdown, but MLV-Luc infection was not significantly inhibited (p>0.05) (Figure 7C). These results confirm the lentiviral specificity of the SET complex.

## Discussion

Our results identify the SET complex as a cytoplasmic barrier to autointegration. Knockdown of 4 SET complex proteins increased autointegration and decreased chromosomal integration. Knockdown of individual SET complex components reduced HIV infection between 3 and 10 fold and increased autointegration approximately 2–3 fold. Moreover, the SET complex proteins SET and NM23-H1 associate with HIV-1 DNA in the cytoplasm. Although there are reports that other DNA repair factors either facilitate or inhibit HIV-1 infection, most of these have been postulated to influence 2-LTR circle formation or 5′ gap repair and to act at a later stage of infection in the nucleus [3], [39]–[43].

A new assay was developed in this study to measure autointegration. Our results verified that HIV autointegration is IN-dependent and that it occurs around the time of reverse transcription. Autointegrants accumulate preferentially in the cytoplasm, suggesting that most autointegration occurs before PIC nuclear import. The auto-PCR assay is semi-quantitative, since the first step involves conventional PCR. Therefore we were unable to use it to quantify the level of autointegration products during HIV infection in comparison to other HIV DNAs, e.g. late RT and 2-LTR circles. We and others [11],[20] have been unable to detect autointegration products by Southern blot using biochemically fractionated PICs in an in vitro assay. HIV infection is relatively inefficient and only autointegration products arising from opposite-strand joining yield a product of discrete size (Figure S4). The frequency of opposite- versus same-strand joining is about 1 to 9 (data not shown), possibly contributing to the difficulty in identifying autointegration products by Southern blot.

The reduction in HIV replication caused by knocking down SET complex genes is lower than the typical log changes seen by inhibiting key viral enzymes, such as reverse transcriptase or IN. This difference is not unexpected, given the postulated role of the SET complex in preventing a suicidal side pathway rather than as an essential host factor in executing viral replication. Moreover, since knockdown is incomplete, the effect we measured might be dampened by the function of the remaining unknocked down protein. This problem could be exacerbated by the fact that the SET complex is very abundant in cells and not much protein would be expected to interact with a single PIC. The observed increase in autointegration and corresponding decrease in chromosomal integration, measured by the auto-PCR and Alu-PCR assays, respectively, were less than the reduction in viral replication as measured by Luc assay or p24 production. These quantitative differences may be due to different sensitivities of the semi-quantitative PCR-based assays as compared to the Luc and p24 assays.

In addition to its role in preventing autointegration, the SET complex might affect other steps in the viral life cycle. For example, SET complex proteins are known to regulate transcription [26],[27] and a slight, but significant, reduction in transcription from a chromatinized HIV reporter gene was observed in SET/NM23-H1 knockdown cells. SET can act as a histone H2B chaperone to either assemble or disassemble nucleosomes, thereby altering accessibility for transcription, and together with pp32 can regulate histone modifications. Moreover, SET, pp32 and NM23-H1 can enhance transcription from some promoters [26],[27].

The SET complex contains 3 DNases - the base excision repair (BER) apurinic endonuclease APE1, a DNA nicking endonuclease NM23-H1, and a 5′-3′ exonuclease TREX1, which may serve as a BER proofreading endonuclease [44]. Our preliminary data suggest that the presumed BER function of the SET complex, which has not formally been demonstrated, is involved in its role in blocking HIV-1 autointegration (data not shown). Although APE1 nicking has previously been proposed as a threat to HIV-1 cDNA [45], our results suggest that within the SET complex, APE1 plays a protective role. One of the roles of BER is to repair misincorporated deoxyuridine in DNA that occurs by utilizing dUTP in place of dTTP or by spontaneous deamination of incorporated cytosines, which is enhanced under oxidative conditions. For HIV-1 this represents a particular problem because reverse transcriptase (RT) does not effectively distinguish dUTP from dTTP and the dUTP/dTTP ratio is especially high in primary immune cells susceptible to HIV-1 infection [46]. Moreover, the host cytidine deaminase APOBEC3G (A3G) can attack the minus strand during reverse transcription in immune cells [47],[48] and introduces dC-to-dU changes that can be repaired by BER. Although most of our results were obtained in A3G− HeLaCD4 cells, we also showed that the SET complex facilitates HIV replication and integration in A3G+ Jurkat T cells. In addition to its nuclease function, NM23-H1 is a nucleoside diphosphate kinase that catalyzes the exchange of dNDPs for dNTPs and therefore potentially regulates the pool of nucleotides available for reverse transcription and/or repair.

HIV autointegration generates defective DNA products including nicked inverted and subgenomic dsDNA circles ([34] and Figure S4). The presence of these DNAs in the cytoplasm can potentially be recognized by cytosolic DNA sensors and trigger the interferon (IFN)-stimulatory DNA (ISD) response [5],[49]. One of the SET complex proteins, TREX1, has recently been identified as a negative regulator of the ISD response [6]. TREX1 is the major 5′-3′ DNA exonuclease in mammalian cells, and mutations in the human TREX1 gene are associated with Aicardi-Goutieres syndrome (AGS), lupus syndromes and other pro-inflammatory autoimmune diseases [50]–[53]. TREX1 deficient cells accumulate cytoplasmic DNA derived from endogenous retroelements [6], which can then activate IRF3 to trigger production of type I IFNs leading to autoimmunity. Endogenous retroelements share many features with retroviruses, including cytoplasmic reverse transcription and chromosomal integration. Retroelements can also undergo autointegration [10]. In this study, TREX1 knockdown inhibited HIV infection 10-fold, representing the strongest effect of any of the SET components tested (Figure 2A). By both reducing autointegration and digesting DNA products produced during the autointegration events that do occur, TREX1 may further promote HIV infection by inhibiting the secretion of Type I IFNs, key effectors of antiviral innate immunity.

We are intrigued by the possibility that HIV nucleic acids may engage similar cell-intrinsic factors as endogenous retroelements. One example of such a factor is A3G, which was identified through its ability to mutate the genome and inhibit HIV infection [48],[54],[55]. APOBEC3 proteins also inhibit Alu and LINE-1 retrotransposition, by potentially sequestering retrotransposon RNAs in high-molecular-weight complexes [54],[56].

Understanding how the SET complex binds to the HIV PIC and regulates lentiviral autointegration requires further study. Viral DNA in the PIC is accessible to exogenously introduced endonucleases [21],[57],[58], so a direct interaction between SET complex proteins and HIV-1 DNA is plausible. We do not know whether the SET complex remains associated with the PIC during and after nuclear import. Since the SET complex shuttles back and forth to the nucleus [22],[26], this remains a distinct possibility. The mechanism used by the SET complex to inhibit lentiviral autointegration may provide insight into how to inhibit viral replication by inducing autointegration. Small molecule drugs that inhibit SET complex function or change its cellular distribution could be explored for antiviral therapy.

## Materials and Methods

### Cell lines

Cells were grown in Dulbecco's modified Eagle's medium (DMEM) (Gibco) supplemented with 10% heat-inactivated fetal bovine serum (FBS) at 37°C and 5% CO_2_ unless specified otherwise. HeLaCD4 and TZM-bl cells were obtained form the NIH AIDS Research and Reference Reagent Program. Jurkat cells, obtained from ATCC, were maintained in RPMI medium supplemented with 10% FBS. MDA-MB-435 cell lines, C100 and H1-117 were a kind gift of Patricia Steeg (NCI) [59]. Chicken DF1 cells (a gift of James Cunningham, Harvard Medical School) were propagated in DMEM/10% FBS, 100 U/mL penicillin G sodium, and 100 µg/mL streptomycin sulfate. The *ledgf* ^+/+^ and *ledgf* ^−/−^ MEF used in this study are described as E17(+/+) and E16(−/−), respectively, in [60].

### Virus production and infection

HIV_IIIB_ was propagated as described previously [61]. HIV-2 strain MLP-15132 was obtained from the NIH AIDS Research and Reference Reagent Program (contributed by Lutz Gürtler and Friedrich Deinhardt). HIV-2 infectivity was measured by FACS staining with p24-FITC antibody (BD Biosciences) 24 hpi. HIV-Luc and MLV-Luc constructs were described previously [18]. SIV-Luc was kindly provided by Nathaniel Landau (NYU). Viral supernatants were produced from transfected 293T cells as described [18]. ASV-Luc (24 mL) was produced from DF1 cells plated at 2×10^6^/10 cm dish the day prior to co-transfecting with 15 µg pRIAS-Luc and 10 µg pHCMV-G [62] using Fugene 6 as recommended by the manufacturer. Virus was harvested over 3 successive days and concentrated approximately 32-fold by ultracentrifugation prior to use. HIV-1 was titered by p24 ELISA, and infections were performed for 6–8 h at an MOI of 1 before replacing viral supernatants with fresh medium. Luc activity was assayed 48 hpi as described [18]. Briefly, cells in 12-well plates were lysed with 250 uL 1× Passive Lysis Buffer (Promega) for 15 min at room temperature. Cell lysates were collected as supernatants after a quick spin to pellet cell debris. Luc activity was measured using Luc Assay Reagent (Promega) substrate in a Synergy 2 luminometer (BioTek). Protein levels in cell lysates were determined by BCA assay (Thermo Scientific). β-galactosidase activity was measured using Gal Screen (TROPIX). shRNA containing lentiviruses were generated by co-transfecting 293T cells with three plasmids, pLentiLox-shRNA [63], pHRgagpol and pVSVG (4∶4∶2 ratio), and viral supernatants were collected 48 h post transfection. Jurkat cell lines expressing shRNAs were generated by infection with VSV-G-pseudotyped lentivirus containing sh-CTL or sh-APE1 and sorting for GFP expressing cells 2 d later. These cells were subsequently challenged with single-round reporter viruses to test the role of APE1 in T cell infection. All Jurkat cell infections were done by spinoculation at 1500 g for 2 h.

For experiments that measured stage-specific HIV-1 DNAs, viral supernatants were pretreated with 40 U/mL Turbo DNase (Ambion) at 37°C for 1 hr. Cells were infected using DNase-treated viruses, and DNA was isolated using the Hirt method [64] at specified times post infection.

### Plasmids, siRNAs, transfection

SET cDNA was PCR amplified from pET26b-SET [22] using primers containing *Bam*HI and *Xho*I restriction sites and a FLAG-HA dual-tag on the C-terminal end ( DYKDDDDKQQYPYDVPDYA , FLAG-QQ-HA). The resultant fragment was subsequently cloned into pcDNA3 (Invitrogen) to generate pcDNA-SET-FLAG-HA for expression in mammalian cells. pcDNA-SET-in-FLAG-HA (insensitive to SET siRNA) was constructed based on pcDNA-SET-FLAG-HA with silent mutations introduced using the QuikChange kit (Statagene). Primers were: forward primer: 5′-CCAAccacgacggCGCGGATGAAACGTCTGAGaaagaacagc-3′; reverse primer: 5′-GCTGTTCTTTCTCAGACGTTTCATCCGCGCCGTCGTGGTTGG-3′.

pRIAS-Luc, which encodes for single-round (replication-incompetent) ASV carrying the Luc reporter gene (ASV-Luc), was built by amplifying Luc sequences from pNLX.Luc(R-) [65] with primers AE675 (5′-GGTACTATCGATAAAGCCACCATGGAAG)/ AE3292 (5′-CTAGATCGATTACACGGCGATCTTTCC), digesting with Cla I, and ligation to Cla I-digested pRIAS [66]. siRNAs were transfected using Oligofectamine (Invitrogen) following manufacturer's protocols. Cells transfected twice in two consecutive days were infected on the third day. DNAs were transfected using Lipofectamine 2000 (Invitrogen) following manufacturer's protocols. siRNAs were purchased from Dharmacon. Catalog numbers are given for siRNAs pre-designed by Dharmacon and a single siRNA that gave the best knockdown from each set of 4 was labeled as ‘preferred’ and used in this study. An equal molar mix of two TREX1 siRNAs was used to maximize knockdown. All sequences correspond to sense strand sequence of the target gene.

CD4 siRNA: 5′-GAUCAAGAGACUCCUCAGU-3′ [67]
SET siRNA: 5′-ggccgacgagaccucagaa-3′ [24]
NM23-H1 siRNA: 5′-GGAUUCCGCCUUGUUGGUC-3′ [24]
TREX1 siRNA: #1 5′-CCAAGACCATCTGCTGTCA-3′; #2 5′-ACAATGGTGACCGCTACGA-3′ [23]
BAF-c siRNA: 5′-GAAGCUGCACGUAAGGGGU-3′ [18]
BAF siRNA: 5′-GAAGCUGGAGGAAAGGGG-3′ [18]
APE1 siRNA (cat# J-010237): #1 5′-CAAAGUUUCUUACGGCAUA-3′ (preferred); #2 5′-GAGACCAAAUGUUCAGAGAUU-3′; #3 5′-CUUCGAGCCUGGAUUAAGA-3′; #4 5′-UAACAGCAUAUGUACCUAA-3′
Non-targeting siRNA #1 (cat# D-001210-01): 5′-UAGCGACUAAACACAUCAA-3′

sh-CTL and sh-APE1 were cloned into pLenti-LOX3.7 using the following oligos:

sh-CTL: 5′-TGTAGCGACTAAACACATCAATTCAAGAGATTGATGTGTTTAGTCGCTACTTTTTTC -3′ and 5′- TCGAGAAAAAAGTAGCGACTAAACACATCAATCTCTTGAATTGATGTGTTTAGTCGCTACA -3′
sh-APE1:5′-TGCAAAGTTTCTTACGGCATATTCAAGAGATATGCCGTAAGAAACTTTGCTTTTTTC-3′ and 5′- TCGAGAAAAAAGCAAAGTTTCTTACGGCATATCTCTTGAATATGCCGTAAGAAACTTTGCA-3′.

### Integration site sequence analysis

Human (build 36.1, UCSC hg18 release) genomic sequence and HIV-1_NL4-3_ sequence databases were used for integration site sequence analysis, which was done as described [29].

### PIC isolation, HIV DNA immunoprecipitation (IP), antibodies

HIV PICs were isolated as described [29],[58] with slight modifications. Briefly, HeLaCD4 cells grown on 10 cm plates (80% confluent) were infected with DNase-treated HIV-Luc. Each plate provided enough cells for two IP experiments. Cells were washed with cold Buffer K−/− (20 mM HEPES, pH 7.6, 150 mM KCl, 5 mM MgCl_2_) twice 6 hpi and lysed by rocking at room temperature for 8 min in 0.5 mL Buffer K+/+ (Buffer K−/− containing 1× Protease inhibitors (EDTA-free, Roche), 0.025% digitonin, 1 mM DTT) per 10 cm plate. Supernatants were obtained following successive centrifugations at 1,500× g for 4 min at 4°C and 15,000× g for 1 min at 4°C. Resultant cytoplasmic PIC extracts were incubated with specific antibodies that were pre-bound to protein A or G agarose beads overnight. Beads were washed the next morning with 100 mM KCl wash buffer twice (20 mM Tris7.4, 0.2 mM EDTA, 100 mM KCl, 5 mM ß-mercaptoethanol, 1× protease inhibitors complete, 10% glycerol) and again with the same buffer containing 300 mM KCl before elution with 2× 100 µL 200 mM glycine (pH 3). Eluates were neutralized by adding 2 µL of 1.5 M Tris-HCl (pH 8.8) before phenol/chloroform/iodoacetamide extraction and DNA precipitation. HIV-1 DNA in the IP was quantified using qPCR with late RT primers (MH531/MH532). Antibodies for IP were: anti-IN (rabbit, affinity purified) [68], anti-MA (mouse 3H7) [69], anti-SET (rabbit, affinity purified) [22], anti-NM23-H1 (rabbit, Santa Cruz #sc343) and anti-LEDGF/p75 (mouse, BD Transduction #611714). Antibodies used for immunoblot were anti-Ape1 (rabbit, this study), anti-BAF (rabbit, a kind gift of Katherine Wilson, John Hopkins University School of Medicine) [70].

### Quantitative PCR, auto-PCR

HIV-1 late RT, integrated DNA, and 2-LTR circles were quantified as previously described [29],[30]. Briefly, mitochondrial DNA, late RT and 2-LTR circles in extrachromosomal DNA fractions were analyzed by qPCR using MIT+/MIT−, MH531/MH532 and AE2948/AE2949 primers, respectively (sequences below). β-globin DNA was similarly measured in chromosomal DNA fractions using β-globin+/β-globin− primers (sequences below). Integrated HIV DNA was also measured in chromosomal fractions, but by Alu-PCR followed by nested qPCR using AE989/AE990 primers (sequences below). Autointegration products were measured using a two-step nested PCR: Step 1 is a semiquantitative PCR using 200 ng extrachromosomal DNA, 1× PCR buffer, 1.5 mM MgCl_2_, 0.2 µM of each primer (PBS−, NY200/A+, NY199/B−), 0.2 mM of each dNTP and 1.5 U Platinum Taq polymerase (Invitrogen) in a 25 µL reaction volume. PCR program was 94°C/5 min, 24 cycles of 95°C/30 s-60°C/30 s-72°C/3 min, then 72°C/7 min. PCR products from Step 1 were diluted 1∶100 for use in Step 2. Step 2 was a qPCR assay using AE989/AE990 primers [29]. Autointegration initiated from the downstream U5 end (Figure S5) was measured similarly, except for replacing the PBS− primer with a Luc+ primer (located at the 3′ end of the Luc gene, adjacent to the right LTR).

Primer sequences:

MIT+: 5′-GACGTTAGGTCAAGGTGTAG-3′
MIT−: 5′-CAACTAAGCACTCTACTCTC-3′
MH531 (late RT forward): 5′-TGTGTGCCCGTCTGTTGTGT-3′
MH532 (late RT reverse): 5′-GAGTCCTGCGTCGAGAGAGC-3′
AE2948 (2-LTR forward): 5′-AACTAGGGAACCCACTGCTTAAG-3′
AE2949 (2-LTR reverse): 5′-TCCACAGATCAAGGATATCTTGTC-3′
β-globin+: 5′-GAAGAGCCAAGGACAGGTAC-3′
β-globin−: 5′-AAGCAATAGATGGCTCTGCC-3′
PBS−: 5′-TTTCCGGTCCCTGTTCGGGCGCCA-3′
Luc+: 5′-CAAGAAGGGCGGAAAGATCGCCGTGT-3′
Alu: 5′-TCCCAGCTACTCGGGAGGCTGAGG-3′
AE989 (R): 5′-TCTGGCTAGCTAGGGAACCCA-3′
AE990 (U5): 5′-CTGACTAGGATGGTCTGAGG-3′
NY199/primer B−: 5′-CTACCTTGTTATGTCCTGCTTG-3′
NY200/primer A+: 5′-CTCTACAGCACTTGGCACTAGC-3′

## Supporting Information

Figure S1 Virions released by control and SET/NM23-H1 knockdown cells are equally infectious. Viral supernatants from control and SET/NM23-H1 knockdown HeLaCD4 cells (24 h post-HIVIIIB-infection) were normalized for p24 content, and an equal amount of virons was applied to TZM-bl cells, which are stably transfected with an LTR-driven Luc reporter gene. Luc activity was measured 48 hpi.
(0.04 MB PDF)

Figure S2 SET knockdown inhibits infection of VSV-G pseudotyped HIV-Luc across a range of multiplicities of infection (MOI). HeLa-CD4 cells were transfected first with CTL or SET siRNA and then 24 h later with vector (V) or SET-in plasmid DNA. Transfected cells were then infected with indicated MOIs 24 h later and Luc activity was measured 48 h post-infection. *, p<0.01 relative to control knockdown. Mean and S.D. from two independent experiments are shown.
(0.04 MB PDF)

Figure S3 The consensus sequence for autointegration is indistinguishable between control and SET/NM23-H1 knockdown cells. Nucleotide frequency at each position is shown as the percent of expected frequency if autointegration were random. Frequencies <70% (red) or >130% (green) of expected (corresponding to p<0.001) are in bold. The position 0 nucleotide is joined to the processed U3 end of the LTR. Nucleotide sequences for positions 0–14 were experimentally determined by sequencing; those for positions −10 to −1 were assumed from the HIV-Luc sequence upstream of the mapped integration sites.
(0.09 MB PDF)

Figure S4 Detailed diagram of auto-PCR assay. Primers PBS−/A+ and PBS−/B amplify same-strand and opposite-strand joining products, respectively, during first-round PCR. The resulting products contain PBS-LTR (U3RU5) sequences, which are measured by second-round nested qPCR using R-U5 primers. Arrowheads, reverse transcript 5′ ends; filled circles, 5′ phosphates attacked by the recessed CA-OH ends during autointegration. The viral DNA ends become joined to these internal sites during CA-OH attack; the structures in brackets are imaginary intermediates to aid visualization of reaction pathways. Open circles, internal 3′ termini resulting from autointegration.
(0.29 MB PDF)

Figure S5 Autointegration from U3 and U5 ends of HIV reverse transcripts have similar kinetics. (A) A diagram of HIV-Luc reverse transcripts with auto-PCR primers used. (B,C) Kinetics of autointegration from U3 (B) and U5 (C) ends. HeLa-CD4 cells were infected with VSV-G pseudotyped HIV-Luc and extrachromsomal DNA was isolated at different times post-infection (as indicated). The PBS(−) primer binds adjacent to the left long terminal repeat (LTR), whereas the Luc(+) primer binds adjacent to the right LTR (the Luc gene replaces nef in the HIV-Luc construct). In the first auto-PCR round, PBS/A/B primers were used to amplify autointegration events initiated through the U3 end (B), and similarly Luc/A/B primers were used to amplify autointegration events initiated through the U5 end (C). The same LTR primers were used in the second-round qPCR.
(0.34 MB PDF)

Figure S6 Knocking down BAF does not affect HIV autointegration. (A) Immunoblot demonstrating knockdown of BAF protein by BAF siRNA but not control siRNA (CTL) or BAF-C siRNA (which contains three mismatches compared to BAF siRNA [1]). By densitometry, only 8% of BAF protein remained at the time cells were infected with HIV-Luc 48 h after transfection. BAF knockdown inhibited HIV infection about 2-fold as measured by Luc activity (B), but had no effect on autointegration (C). Infectivity and autointegration were measured as in Figure 6. Mean and S.D. of two independent experiments are shown. 1. Shun MC, Daigle JE, Vandegraaff N, Engelman A (2007) Wild-type levels of human immunodeficiency virus type 1 infectivity in the absence of cellular emerin protein. J Virol 81: 166–172.
(0.08 MB PDF)

Table S1 Autointegrant sequences recovered from WT and mt IN viral infections. *Clones that did not contain any viral sequence are not shown. †The CA dinucleotide at the end of the cleaved U3 minus strand is underlined. ‡Number refers to position in reference HIV-1NL4-3 strain.
(0.05 MB PDF)
