# Supplementary material for: The SET Complex Acts as a Barrier to Autointegration of HIV-1
Source: PLoS Pathog. 2009 Mar 6;5(3):e1000327. doi: 10.1371/journal.ppat.1000327 (PMC2644782; doi:10.1371/journal.ppat.1000327)
Supplement: Figure S1 — Virions released by control and SET/NM23-H1 knockdown cells are equally infectious. Viral supernatants from control and SET/NM23-H1 knockdown HeLaCD4 cells (24 h post-HIVIIIB-infection) were normalized for p24 content, and an equal amount of virons was applied to TZM-bl cells, which are stably transfected with an LTR-driven Luc reporter gene. Luc activity was measured 48 hpi. (0.04 MB PDF) [file ppat.1000327.s001.pdf]

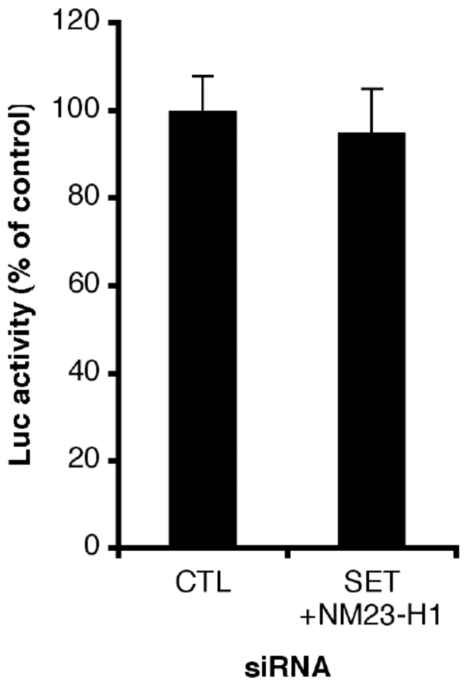

Figure S1. Virions released by control and SET/NM23-H1 knockdown cells are equally infectious. Viral supernatants from control and SET/NM23-H1 knockdown HeLaCD4 cells (24 h post HIV_{IIIB} infection) were normalized for p24 content and an equal amount of virons was applied to TZM-bl cells, which are stably transfected with an LTR-driven Luc reporter gene. Luc activity was measured 48 hpi.
