# Supplementary material for: The SET Complex Acts as a Barrier to Autointegration of HIV-1
Source: PLoS Pathog. 2009 Mar 6;5(3):e1000327. doi: 10.1371/journal.ppat.1000327 (PMC2644782; doi:10.1371/journal.ppat.1000327)
Supplement: Figure S2 — SET knockdown inhibits infection of VSV-G pseudotyped HIV-Luc across a range of multiplicities of infection (MOI). HeLa-CD4 cells were transfected first with CTL or SET siRNA and then 24 h later with vector (V) or SET-in plasmid DNA. Transfected cells were then infected with indicated MOIs 24 h later and Luc activity was measured 48 h post-infection. *, p<0.01 relative to control knockdown. Mean and S.D. from two independent experiments are shown. (0.04 MB PDF) [file ppat.1000327.s002.pdf]

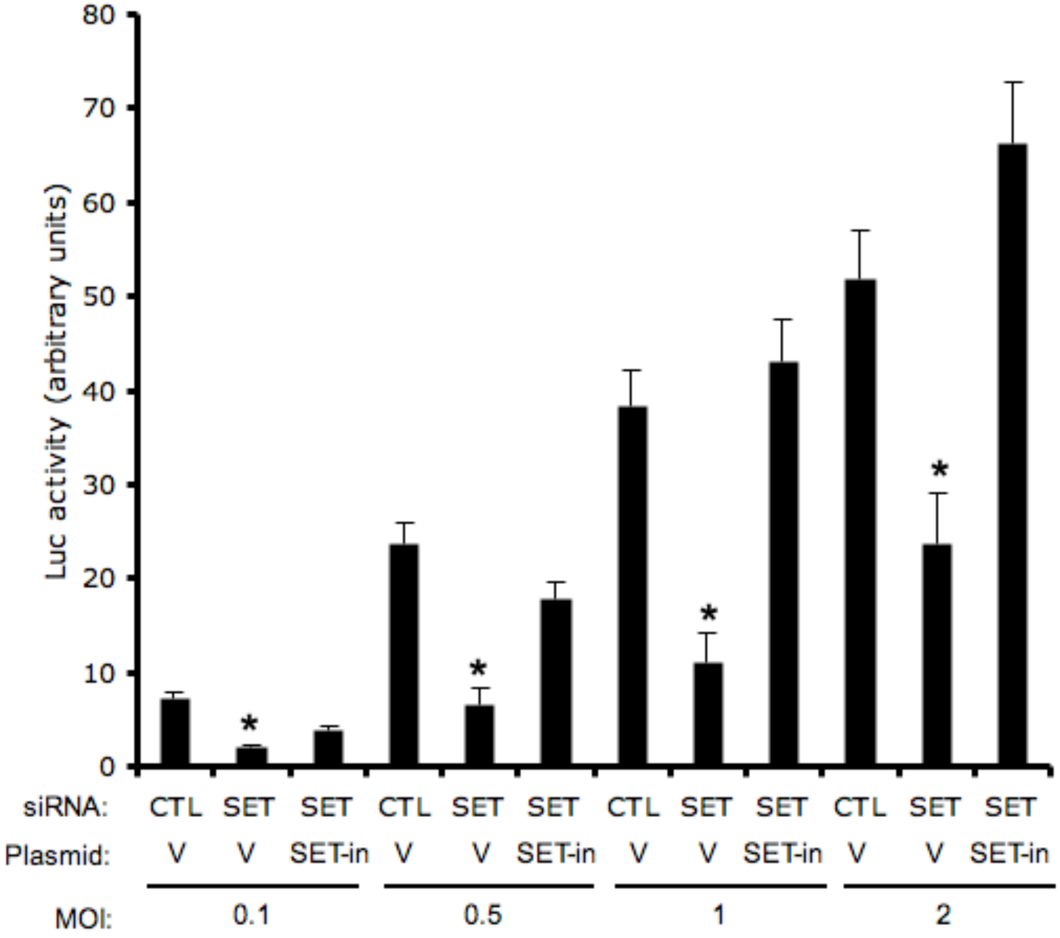

Figure S2. SET knockdown inhibits infection of VSV-G pseudotyped HIV-Luc across a range of multiplicities of infection (MOI). HeLa-CD4 cells were transfected first with CTL or SET siRNA and then 24 h later with vector (V) or SET-in plasmid DNA. Transfected cells were then infected with indicated MOIs 24 h later and Luc activity was measured 48 h post infection. *, $p<0.01$ relative to control knockdown. Mean and S.D. from 2 independent experiments are shown.
