# Supplementary material for: The SET Complex Acts as a Barrier to Autointegration of HIV-1
Source: PLoS Pathog. 2009 Mar 6;5(3):e1000327. doi: 10.1371/journal.ppat.1000327 (PMC2644782; doi:10.1371/journal.ppat.1000327)
Supplement: Figure S3 — The consensus sequence for autointegration is indistinguishable between control and SET/NM23-H1 knockdown cells. Nucleotide frequency at each position is shown as the percent of expected frequency if autointegration were random. Frequencies <70% (red) or >130% (green) of expected (corresponding to p<0.001) are in bold. The position 0 nucleotide is joined to the processed U3 end of the LTR. Nucleotide sequences for positions 0–14 were experimentally determined by sequencing; those for positions −10 to −1 were assumed from the HIV-Luc sequence upstream of the mapped integration sites. (0.09 MB PDF) [file ppat.1000327.s003.pdf]

Control siRNA knockdown autointegration sites

position:	-10	-9	-8	-7	-6	-5	-4	-3	-2	-1	**0**	**1**	**2**	**3**	**4**	5	6	7	8	9	10	11	12	13	14
**A**	123	119	112	123	116	83	94	80	98	123	105	112	137	156	**62**	90	112	**156**	116	145	137	101	109	119	127
**T**	123	101	117	107	110	126	114	**166**	150	120	**37**	160	126	117	98	126	129	77	86	120	101	132	138	120	107
**G**	87	149	97	97	103	138	154	118	159	128	**241**	87	87	87	113	87	**77**	97	128	103	159	92	118	113	133
**C**	133	114	148	148	148	133	123	94	**54**	99	148	89	109	99	**232**	**173**	148	153	163	94	79	143	99	118	109

SET/NM23-H1 siRNA knockdown autointegration sites

position:	-10	-9	-8	-7	-6	-5	-4	-3	-2	-1	**0**	**1**	**2**	**3**	**4**	5	6	7	8	9	10	11	12	13	14
**A**	104	94	102	89	92	64	89	67	77	109	87	92	74	139	**35**	79	114	146	94	127	131	127	97	79	112
**T**	98	110	92	98	128	106	108	135	120	79	**24**	139	112	106	79	84	120	55	86	67	69	86	104	100	100
**G**	112	89	92	109	92	129	89	119	172	155	**205**	79	112	73	125	73	**40**	86	106	96	99	76	125	122	86
**C**	87	102	117	108	75	108	111	72	**33**	69	132	72	102	69	**186**	**174**	108	123	123	120	108	111	75	105	99

Figure S3. The consensus sequence for autointegration is indistinguishable between control and SET/NM23-H1 knockdown cells. Nucleotide frequency at each position is shown as the percent of expected frequency if autointegration were random. Frequencies <70% (red) or >130% (green) of expected (corresponding to p<0.001) are in bold. The position 0 nucleotide is joined to the processed U3 end of the LTR. Nucleotide sequences for positions 0-14 were experimentally determined by sequencing; those for positions -10 to -1 were assumed from the HIV-Luc sequence upstream of the mapped integration sites.
