# Supplementary material for: The SET Complex Acts as a Barrier to Autointegration of HIV-1
Source: PLoS Pathog. 2009 Mar 6;5(3):e1000327. doi: 10.1371/journal.ppat.1000327 (PMC2644782; doi:10.1371/journal.ppat.1000327)
Supplement: Figure S4 — Detailed diagram of auto-PCR assay. Primers PBS−/A+ and PBS−/B amplify same-strand and opposite-strand joining products, respectively, during first-round PCR. The resulting products contain PBS-LTR (U3RU5) sequences, which are measured by second-round nested qPCR using R-U5 primers. Arrowheads, reverse transcript 5′ ends; filled circles, 5′ phosphates attacked by the recessed CA-OH ends during autointegration. The viral DNA ends become joined to these internal sites during CA-OH attack; the structures in brackets are imaginary intermediates to aid visualization of reaction pathways. Open circles, internal 3′ termini resulting from autointegration. (0.29 MB PDF) [file ppat.1000327.s004.pdf]

Yan et al. Figure S4

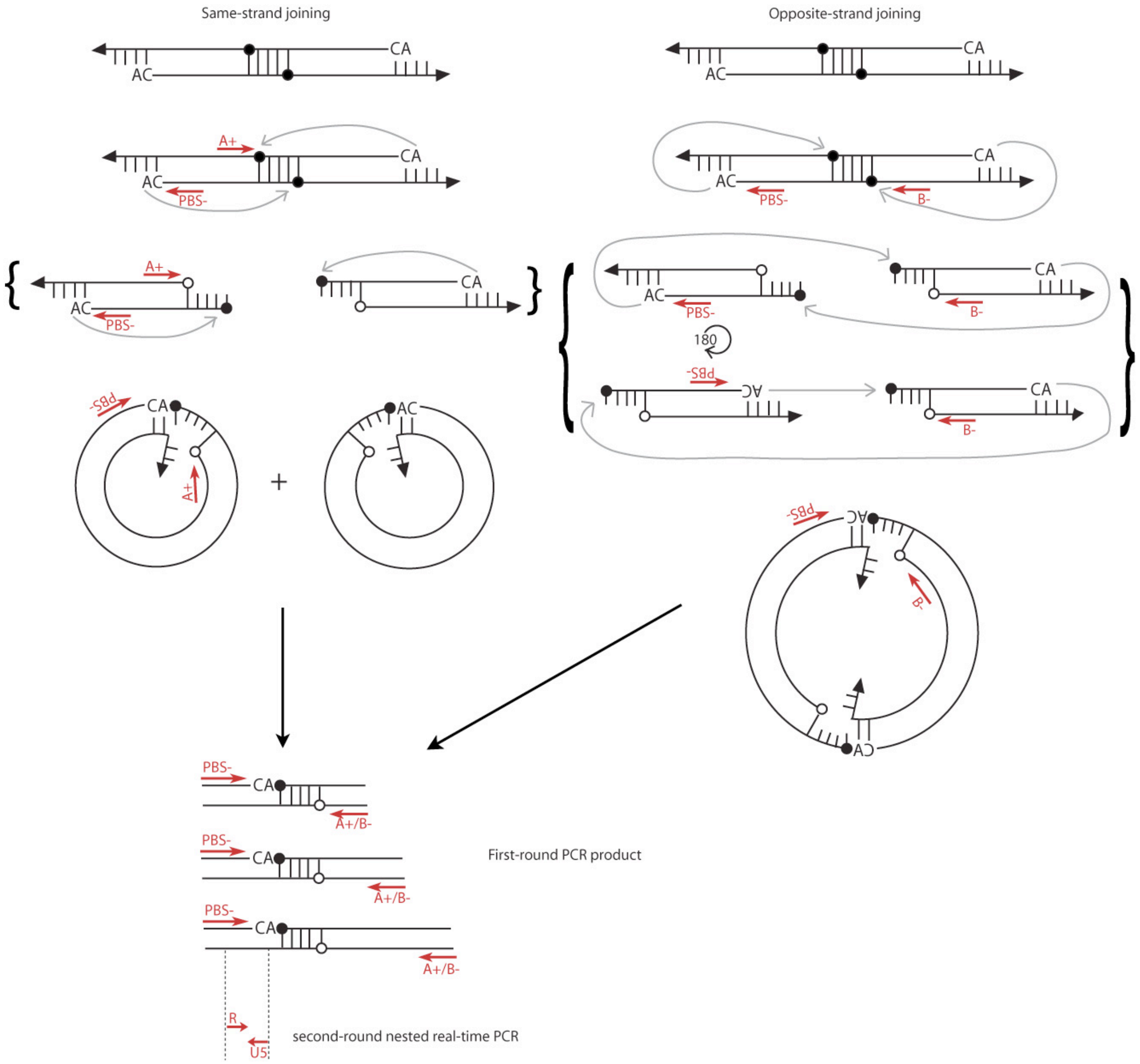

Figure S4. Detailed diagram of auto-PCR assay. Primers PBS-/A+ and PBS-/B- amplify same-strand and opposite-strand joining products, respectively, during first-round PCR. The resulting products contain PBS-LTR (U3RU5) sequences, which are measured by second-round nested qPCR using R-U5 primers. Arrowheads, reverse transcript 5' ends; filled circles, 5' phosphates attacked by the recessed CA-$_{OH}$ ends during autointegration. The viral DNA ends become joined to these internal sites during CA-$_{OH}$ attack; the structures in brackets are imaginary intermediates to aid visualization of reaction pathways. Open circles, internal 3' termini resulting from autointegration.
