# Supplementary material for: The SET Complex Acts as a Barrier to Autointegration of HIV-1
Source: PLoS Pathog. 2009 Mar 6;5(3):e1000327. doi: 10.1371/journal.ppat.1000327 (PMC2644782; doi:10.1371/journal.ppat.1000327)
Supplement: Figure S5 — Autointegration from U3 and U5 ends of HIV reverse transcripts have similar kinetics. (A) A diagram of HIV-Luc reverse transcripts with auto-PCR primers used. (B,C) Kinetics of autointegration from U3 (B) and U5 (C) ends. HeLa-CD4 cells were infected with VSV-G pseudotyped HIV-Luc and extrachromsomal DNA was isolated at different times post-infection (as indicated). The PBS(−) primer binds adjacent to the left long terminal repeat (LTR), whereas the Luc(+) primer binds adjacent to the right LTR (the Luc gene replaces nef in the HIV-Luc construct). In the first auto-PCR round, PBS/A/B primers were used to amplify autointegration events initiated through the U3 end (B), and similarly Luc/A/B primers were used to amplify autointegration events initiated through the U5 end (C). The same LTR primers were used in the second-round qPCR. (0.34 MB PDF) [file ppat.1000327.s005.pdf]

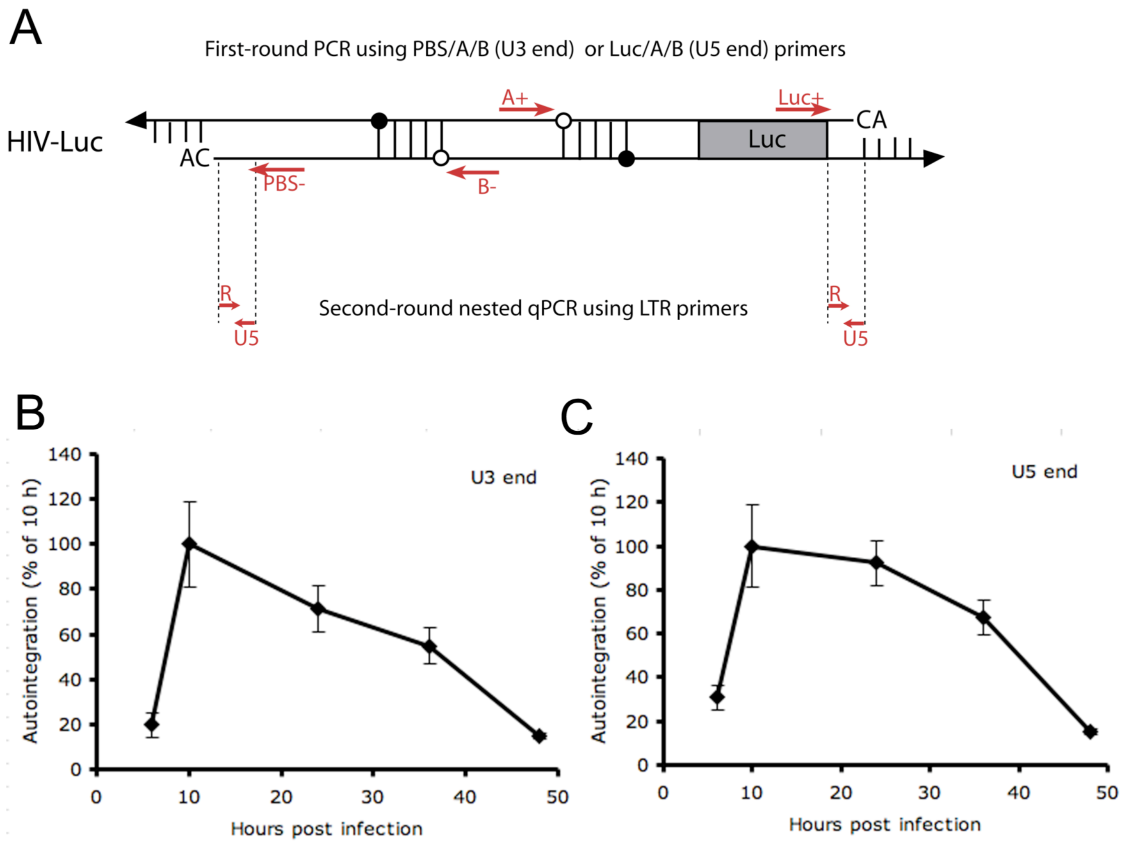

Figure S5. Autointegration from U3 and U5 ends of HIV reverse transcripts have similar kinetics. (A) A diagram of HIV-Luc reverse transcripts with auto-PCR primers used. (B-C) Kinetics of autointegration from U3 (B) and U5 (C) ends. HeLa-CD4 cells were infected with VSV-G pseudotyped HIV-Luc and extrachromsomal DNA was isolated at different times post infection (as indicated). The PBS(-) primer binds adjacent to the left long terminal repeat (LTR) whereas the Luc(+) primer binds adjacent to the right LTR (the Luc gene replaces *nef* in the HIV-Luc construct). In the first auto-PCR round, PBS/A/B primers were used to amplify autointegration events initiated through the U3 end (B), and similarly Luc/A/B primers were used to amplify autointegration events initiated through the U5 end (C). The same LTR primers were used in the second round qPCR.
