# Supplementary material for: The SET Complex Acts as a Barrier to Autointegration of HIV-1
Source: PLoS Pathog. 2009 Mar 6;5(3):e1000327. doi: 10.1371/journal.ppat.1000327 (PMC2644782; doi:10.1371/journal.ppat.1000327)
Supplement: Figure S6 — Knocking down BAF does not affect HIV autointegration. (A) Immunoblot demonstrating knockdown of BAF protein by BAF siRNA but not control siRNA (CTL) or BAF-C siRNA (which contains three mismatches compared to BAF siRNA [1]). By densitometry, only 8% of BAF protein remained at the time cells were infected with HIV-Luc 48 h after transfection. BAF knockdown inhibited HIV infection about 2-fold as measured by Luc activity (B), but had no effect on autointegration (C). Infectivity and autointegration were measured as in Figure 6. Mean and S.D. of two independent experiments are shown. 1. Shun MC, Daigle JE, Vandegraaff N, Engelman A (2007) Wild-type levels of human immunodeficiency virus type 1 infectivity in the absence of cellular emerin protein. J Virol 81: 166–172. (0.08 MB PDF) [file ppat.1000327.s006.pdf]

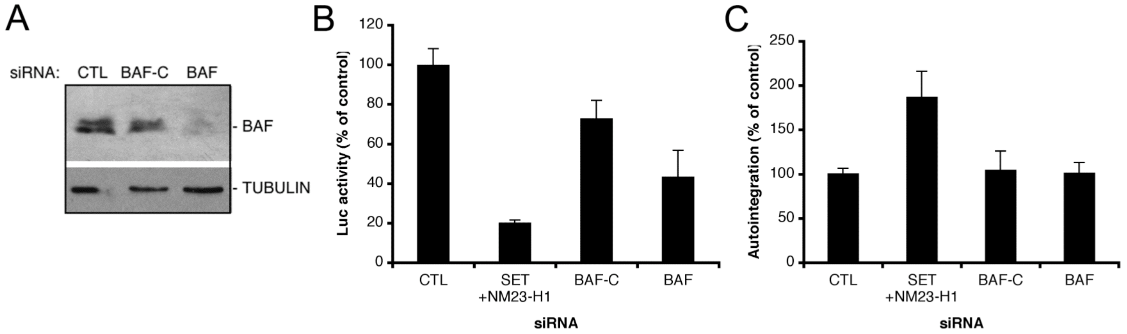

Figure S6. Knocking down BAF does not affect HIV autointegration. (A) Immunoblot demonstrating knockdown of BAF protein by BAF siRNA but not control siRNA (CTL) or BAF-C siRNA (which contains three mismatches compared to BAF siRNA [1]). By densitometry only 8% of BAF protein remained at the time cells were infected with HIV-Luc 48 h after transfection. BAF knockdown inhibited HIV infection about 2-fold as measured by Luc activity (B), but had no effect on autointegration (C). Infectivity and autointegration were measured as in Fig 6. Mean and S.D. of two independent experiments are shown.

1. Shun MC, Daigle JE, Vandegraaff N, Engelman A (2007) Wild-type levels of human immunodeficiency virus type 1 infectivity in the absence of cellular emerin protein. J Virol 81: 166-172.
