# Supplementary material for: The SET Complex Acts as a Barrier to Autointegration of HIV-1
Source: PLoS Pathog. 2009 Mar 6;5(3):e1000327. doi: 10.1371/journal.ppat.1000327 (PMC2644782; doi:10.1371/journal.ppat.1000327)
Supplement: Table S1 — Autointegrant sequences recovered from WT and mt IN viral infections. *Clones that did not contain any viral sequence are not shown. †The CA dinucleotide at the end of the cleaved U3 minus strand is underlined. ‡Number refers to position in reference HIV-1NL4-3 strain. (0.05 MB PDF) [file ppat.1000327.s007.pdf]

Tabel S1. Autointegrant sequences recovered from WT and mt IN viral infections*.

Clone #	DNA junction sequence†	Viral internal insertion site‡	Comment
WT IN autointegrants			
1-2	…TTAGCCTTTCCACCTACAATCC…	4654	U3-internal
1-5	…TTAGCCCTTCCAAAAGATTAGT…	5105	U3-internal
1-7	…TTAGCCCTTCCAGGAGAAAGAG…	5263	U3-internal
1-8	…TTAGCCCTTCCAGTCTCCATAG…	5293	U3-internal
1-9	…TTAGCCCTTCCAAAAGCAAAGA…	5019	U3-internal
1-14	…TTAGCCCTTCCAGAAAGTACTA…	5173	U3-internal
1-15	…TTAGCCCTTCCACTAATCCAAA…	5180	U3-internal
1-16	…TTAGCCCTTCCAGGAGAAAGAG…	5263	U3-internal
1-19	…TTAGCCCTTCCATATAGTTAGT…	5421	U3-internal
1-21	…TTAGCCCTTCCAGGCAGTAGTA…	4970	U3-internal
1-23	…TTAGCCCTTCCAAAGTAGACCC…	5330	U3-internal
1-24	…TTAGCCCTTCCAGTCTCCATAG…	5293	U3-internal
1-27	…TTAGCCCTTCCAGTAATAACAA…	5233	U3-internal
1-3	…TGAATAAAGA…AACAAGGTAG…	4690-5467	Internal only
1-12	…TCCTCTGGAA…AACAAGGTAG…	4951-5467	Internal only
1-30	…ACTAATCCAA…AACAAGGTAG…	5179-5467	Internal only
1-1	LTR only	n/a	
1-6	LTR only	n/a	
1-10	LTR only	n/a	
1-13	LTR only	n/a	
1-20	LTR only	n/a	
1-22	LTR only	n/a	
1-25	LTR only	n/a	
1-29	LTR only	n/a	
mt IN autointegrants			
2-17	…GGGCAAGAAA…AACAAGGTAG…	4509-5467	Internal only
2-4	LTR only	n/a	
2-8	LTR only	n/a	
2-10	LTR only	n/a	
2-18	LTR only	n/a	

*Clones that did not contain any viral sequence are not shown.

†The CA dinucleotide at the end of the cleaved U3 minus strand is underlined.

‡Number refers to position in reference HIV-1_{NL4-3} strain.
